# What Is a Formulary, Anyway? Part 2

**DOI:** 10.3390/pharmacy6030072

**Published:** 2018-07-23

**Authors:** Richard H. Parrish

**Affiliations:** 1 Department of Pharmacy Services, St. Christopher’s Hospital for Children, 160 East Erie Avenue, Philadelphia, PA 19134, USA; Richard.Parrish@americanacademic.com; Tel.: +1-(215)-427-5317; 2 School of Pharmacy, Virginia Commonwealth University, Richmond, VA 23284, USA

**Keywords:** formulary, clinical pharmacy, P&T committee, rational drug therapy

## Abstract

This paper is a continuation of the first paper on drug formularies. It details the various types of formularies, and speculates on the future of formularies as an instrument for improving rational drug therapy.

## 1. Types and Definitions of Formularies

### 1.1. Open Formulary

As used in the literature, the idea of an open formulary [1] could be described as the least structured, least rigorous, most subjective, and with the greatest ease of listing, where pharmaceuticals are authorized primarily through the act of writing a medication order. Moreover, an open formulary implies that no systematic evaluation occurs for a drug to be authorized, i.e., if approved by FDA, it can be used. An open formulary has been described as an oxymoron [2]. Permissive and market-driven, an open formulary represents an indiscriminate listing of pharmaceuticals by therapeutic class, alphabetic brand name, and/or alphabetic generic name.

An open formulary is, in essence, a passive process. Drugs are procured and administered on the demands of individual prescribers. Since the prescribing process is not well understood [3,4] (except perhaps by the pharmaceutical industry), the rationale for medication use is often left to chance at worst, and semi-structured intentions at best. Politically speaking, an open formulary can signify to prescribers and industry the “open mindedness” of the P&T function that operates on an anything goes-cavalier-premise. In reference to a formulary, the modifier, “open”, has no valid meaning. It does not contribute to or clarify the therapeutic nature of pharmacotherapy. It constitutes the universe of marketed products.

### 1.2. Closed Formulary

The converse side of the formulary spectrum often is called a closed formulary. While having the appearance of active, objective review of drug products, the operational definition of a closed formulary could be equated with the pragmatic axiom, “our minds are made up, do not confuse us with the facts”. Many revel in the intellectual delight emanating from the announcement that their formulary is closed. By definition, any formulary is a limited listing, but, to some, a closed formulary implies therapeutic stagnation. In the past, the pharmaceutical industry applied various tactics and strategies—including *ad hominem* attacks on the reputations of committee members—to focus and manipulate the outcomes of any organized evaluative process as such [2]. Today, industry acquiesces to the decisions of expert panels, and attempts to navigate through the process as a condition of participation in the business environment.

A closed formulary denotes a tighter control of the availability of pharmaceutical products on the basis of therapeutic class. Antibiotics, for instance, are evaluated and compared for spectrum of coverage, side effects, dosage forms, and cost. Preferred agents may be identified within subclasses, like aminoglycosides, cephalosporins, fluoroquinolones, and extended spectrum penicillins. Moreover, therapeutic interchange decisions might be made for subdivisions within subclasses, such as the selection of cefotaxime as the third generation injectable cephalosporin of choice. All other possible choices, i.e., ceftriaxone, cefoperazone, and ceftizoxime, are equated by dose and frequency to the selected agent. In this way, the formulary attempts to stimulate price competition by the principle of interchangeability. After establishing criteria for interchangeability, often, partnering relationships are sought with manufacturers who share in the mission of the hospital, health system, or PBM. In this light, the closure of a formulary occurs from a bottom-up or class perspective, rather than top-down. The idea of closed formularies, in operation, may be useful if it identifies those therapeutic categories amenable to therapeutic interchange [5,6]. However, since a name for this process, therapeutic interchange, exists already, the addition of “closed” to “formulary”, is redundant.

### 1.3. Other Types of Formularies

While “open” and “closed” formularies typically are used to denote the spectrum of evaluation, from a passive to active process, other permutations of formularies are known to exist. Limited list, market, restricted, negative, hybrid, preferred list, and incentive-based have been used in the literature to modify further the meaning and definition of a formulary. However, like their open and closed counterparts, these descriptors of formulary do not expand its understanding or application. Limited, restricted, negative, and preferred may connote closed, while market, hybrid, and incentive-based are subsets of open. These adjectives muddle the meaning and functions of a formulary as a methodology for identifying agents of choice.

Formularies establish prescriptive norms and improve quality by optimizing the selection of agents with the highest therapeutic value at the lowest possible cost. In the hospital or health system, drug formularies serve the purposes of minimizing variation and improving the level of prescribing performance. Prescribing non-formulary drugs in this environment increases the hospital’s risk for decreasing contribution margin. From an MCO or PBM perspective, identification of therapeutic categories with product redundancy affords the opportunity to apply a set of interchange rules in an effort to reduce treatment variation. In the case of an individual prescriber and patient, the patient assumes a greater risk for both treatment failure and increased cost through higher copays if non-formulary drugs are prescribed. Many formularies of MCOs, PBMs, and, to some extent, hospitals, are nothing more than an enumeration of reimbursable products that define levels of payment responsibilities.

## 2. Methods for Value Calculation in Pharmacoeconomics

Decision analysis in pharmacoeconomic studies utilizes a tree of alternatives and the probability for achieving predetermined outcomes for a given patient population or sample to determine total costs of treatment. For example, a population of post-operative hip replacement patients requiring VTE prophylaxis is studied for costs and outcomes in a sample of patients. Several common economic value calculation methodologies are been present in the literature over the last 30 years. These include the following: (1) cost minimization analysis (CMA); (2) cost utility analysis (CUA); (3) cost benefit analysis (CBA); and, particularly, (4) cost effectiveness analysis (CEA) [7].

From a market perspective, formularies address the principle of economic calculation and interchangeability. However, consider the following situation. Let’s say that the informed intermediary function of the current prescribing and dispensing process in the United States was suspended. Patients could purchase medications directly from any source of their choosing. The Federal role, in legitimizing drug products and labeling, was scaled back to its pre-1950s context, that is to say, before passage of the Durham-Humphrey amendments to the 1906 Pure Food and Drugs Act. What would happen if the pharmaceutical market was allowed to become normal, essentially devoid of solicited or unsolicited third party intervention?

To answer this question adequately is the subject of a lengthy treatise involving additional questions in the areas of patents, copyrights, and other property issues [8]. Suffice it to say that formularies would be indispensable. Because prices drive normal markets, comparative product evaluation would become the prime operational business norm, which would allow buyers and patients better value information and leverage. In this environment, formularies would become truly pharmacopedic in nature, and not merely administrative instruments that sanction reimbursement. Increased competition would stimulate research and development because companies and providers would have no incentive to remain stagnant or complacent with current market conditions. Rather, they would seek other real competitive advantages from further development of existing products and extension of product lines. True system transformation and economic expansion would have the simultaneous effect of lowering price, increasing quantity demanded, and improving the quality of supply. Price controls would not inhibit any provider from charging what the market would bear.

In the Canadian province of Alberta, the government allocates tax revenues through Alberta Health (AH) to Alberta Health Services (AHS) and Alberta Blue Cross (ABC). The decision to allow the importation of drugs across the national border is adjudicated through Health Canada. Alberta Blue Cross (ABC) determines the reimbursement decision in the outpatient setting, AHS manages the formulary within its own auspices, and a major part of this decision-making is driven through the value calculations derived from ABC’s determination. Specialized access to high cost drugs is managed through a special AH account, called the Specialized High Cost Drug Program, a provincial cabinet-level financed activity [9]. The cost of these medications is managed through a series of iterations involving the clinical and economic justification for an individual patient’s situation. Prescription medications are available from a number of providers, and some are pharmacist-only medications. In addition, every clinical pharmacist in the province has some level of prescriptive authorization called adaptation, and many have additional prescribing authorization (APA) that improves access to care, especially in rural areas, as recent studies there have demonstrated [10,11]. The financing mechanism changed recently from a generic drug-dispensing rebate to two specific patient care functions that pharmacists can perform: (1) Comprehensive Annual Care Plan; or (2) Standard Medication Management Assessment. Until last year, pharmacists with APA were allowed to charge AH and/or ABC at a higher rate than non-APA pharmacists. The AH website lists nine other direct patient care activities that pharmacists can bill the government [12]. Since 2007, pharmacists in the province have been able to initiate, modify, and discontinue drug therapy in collaboration with and pursuant to an established medical diagnosis [13]. As one that has practiced in this environment from 2013 to 2016, the level of professional practice authority and responsibility, as codified in statute and health acts, are both challenging and unequaled in Canada or internationally. We should know more about the safety and effectiveness trends in Alberta in the next several years, as the Auditor General of Alberta has signaled the need for studies that document the relative success of pharmacist care planning efforts [14].

## 3. Outlook for Objective Review and Evaluation

One view about formulary design in early discussions about formularies in the 1980s was that the existence of a formulary is evidence of an explicit failure of public policy to require and incorporate comparative studies of relative safety and effectiveness prior to the approval of a new pharmaceutical entity [15]. If the criteria of economic calculation and interchangeability were applied by governments, like in Canada and the European Union, let’s say, to computers or retirement financing schemes and products, the result may delineate products of choice, but could eliminate other products at the margin. One could not afford the price of a high-end computer or live on the return from a low-end investment product. These fallacies stem, as Hazlett so clearly observed, from one of two central fallacies, or both, and these include (1) looking only at the immediate consequences of an act or proposal, and (2) looking at the consequences only for a particular group to the neglect of other groups [16]. The price system works to the extent that value calculations are allowed to fluctuate; if demand falls off for some product, its price and the profit in making it go lower, and its production declines [16]. The operation and reality of any expanding economic market happens at the margins, not in the middle or the average of the distribution. Trading goods and services happens because each party expects to be better off in the future as a result of the exchange. Value calculation is required in all forms of value trading which, in the case of prescription drugs, is performed by either providers and/or payers, neither of which is the ultimate consumer of the good. As previously stated, the pharmaceutical market is not a normal market. The promise of the field of pharmacoeconomics lies in studies that essentially demonstrate the value of using pharmaceuticals within various treatment contexts. In other words, there is a compelling biological purpose in pharmaceutical utilization; value implies the question, *of value to whom and for what purpose*? Questions of value are better left to parties operating at the point-of-prescribing level. Formularies that increase the ability of these parties to cope in autonomy may be the most successful ones. In the final analysis, the term, “formulary”, in the current sense of its application is largely pejorative, and additional modifiers and descriptions tacked onto it only magnify the political nature of drug selection within a given environment. A pharmacopeia, defined in Dorland, is an authoritative treatise on drugs and their properties, i.e., a book containing a list of products used in medicine, with descriptions, chemical tests for determining identity and purity, and formulae for certain mixtures of these substances. It generally contains a statement of average dose (the publication of) drugs that are worthy of recognition [17].

A pharmacopeia more accurately describes the outcome of organized drug product selection efforts, and can remedy the information inequities when the producer controls all of the known facts about their drug products. In this context, modifiers are used to further delineate areas of specialization within medical care, i.e., pediatric pharmacopeia, etc., or disease state like diabetes pharmacopeia rather than to indicate the degree of bureaucratic machinations, i.e., closed or open, or site of application like hospital or PBM. The essential question then becomes, how do we employ pharmacotherapy in the treatment of X disease or condition? In the wider scope, these treatment patterns become, for example, “the HCA way” or “the Merck way” or “the Singapore way” for rendering care to patients’ particular medical problems. The clinical utility of the pharmacopeia would be judged in the context of broader therapeutic objectives with future interest directed at improving the allocation of resources for producing cure, prophylaxis, palliation, and management of diseases.

The future of formularies, as judged by the current state of medical treatment and reimbursement, will continue on an unabated, fractional trajectory until producers and payers realize two essential elements of systematic malfunction. The first, a public policy issue, pertains to access, and the second, a clinical issue, entails the nature of demand for care. In the first case, eliminating arbitrary barriers to competition improves health care access. Secondly, the law of demand makes explicit the future of formularies in the context of value trading; *the demand for A is the demand for A*. A normal market where prices are derived from free trade and mutual consent benefits everyone, however not necessarily to the same extent. It optimizes the opportunity to relieve human suffering and solve clinical problems, stimulates innovation, and raises the general standard of living.

I know that I have presented quite controversial ideas about the value of formularies, and identified the circumstances where I believe their societal benefit can be realized more completely. It is, however, a minority opinion. I welcome your critiques, questions, and ideas about removing barriers to health care and improving the general level of quality that each patient should expect when visiting any health care provider or taking any pharmaceutical product.
